# Development and evaluation of the COntextualised and Personalised Physical activity and Exercise Recommendations (COPPER) Ontology

**DOI:** 10.1186/s12966-025-01744-5

**Published:** 2025-05-05

**Authors:** M. Braun, S. Carlier, A. De Paepe, F. De Backere, F. De Turck, G. Crombez

**Affiliations:** 1 Department of Clinical Experimental and Health Psychology, Ghent University, Ghent, Belgium; 2 Institute for Implementation Science in Health Care, University of Zurich, Zurich, Switzerland; 3 Department of Information Technology, Ghent University – imec, Ghent, Belgium

## Abstract

**Background:**

Personalised recommendations for action and coping plans for physical activity (PA) may reduce user burden and increase plan quality. Ontologies are a promising alternative to existing black-box approaches for creating such personalised recommendations as they are able to integrate knowledge from domain experts, input from end-users and data. Here, we report the development of an ontology of physical activities action and coping plans relevant for primary prevention.

**Methods:**

Ontology specification was carried out using literature research, requirement analysis using use case scenarios, and decision-tree workshops. Conceptualisation combined input from existing theories and classification systems, end-users, domain experts and data sets to create lists of concepts, labels, definitions, properties and relationships. Logic rules were created during ontology formalization, and the entire ontology was translated into Web Ontology Language using Protégé. The ontology was checked for logical consistency. The process was evaluated using the Open Biomedical and Biological Ontology (OBO) Repository Principles, and the resulting recommendations using competency questions and use cases.

**Results:**

The ontology consists of an upper-level ontology, and lower-level ontologies for personal profile, planning, activity, context, barrier, and coping strategy. The final ontology consists of 288 classes, 9 data properties and 64 object properties. Development followed OBO ontology design principles. The ontology is logically and structurally consistent, and resulting recommendations were deemed relevant based on competency questions and use cases.

**Conclusions:**

This is the first ontology focusing on physical activity that (1) follows OBO design principles, including being openly available, (2) includes profile and context information and (3) maps knowledge regarding barriers and coping strategies. It can be used as the base of decision-support systems for action and coping planning for physical activity in primary prevention in Western-European adults, and is easily adaptable to other target groups. Challenges and opportunities of ontologies in health promotion are discussed.

**Supplementary Information:**

The online version contains supplementary material available at 10.1186/s12966-025-01744-5.

## Background

A theory-based approach to promote physical activity (PA) is to support users through a planning process, including planning what to do, where, when and with whom (action planning) and anticipating possible barriers and ways to cope with them (coping planning) [1, 2]. These planning-based interventions aim to bridge the gap between intention and behaviour, and have shown to be effective in promoting a range of health behaviours [3, 4] including physical activity [5]. However, long-term usage of planning-based interventions is low [6, 7]. Receiving support in creating action- and coping plans has been mentioned by users as a way to make applications more acceptable and user-friendly [8], as making plans from scratch is often experienced as effortful by users. Also, providing recommendations has the potential to improve the quality of plans which can be low in user-created plans [9].

One way to provide personalised support is through face-to-face contact, for example by personal trainers or healthcare professionals, who rely on their academic knowledge and experience. This process is time- and resource-intensive and not easily scalable. Also, data-driven techniques, such as recommender systems can provide recommendations, usually based on similar individuals or an individual’s own past behaviour [10]. While such systems are good at providing recommendations that users will like and follow, they do not contain any rationale in their recommendations [10]. It is also impossible to disentangle the processes that led to specific recommendations, referred to as a black-box approach [11–13]. Such recommender systems can lead to negative patterns of behaviours being reinforced. The lack of transparency makes it also difficult to detect these patterns and intervene on them. An example of this could be that trends existing in data, such as women doing more household tasks [14, 15], will be taken over into recommendations, thereby reinforcing existing patterns.

A promising alternative to recommender systems to integrate conceptual and theoretical knowledge are ontologies. An ontology is a formal, explicit specification of a shared conceptualisation of a domain of interest [16]. In simpler terms, an ontology is a set of concepts and categories that represent the knowledge of a particular domain, as well as the relationships between those concepts and categories. Ontologies are often represented in a machine-readable format, which allows computers to reason about the concepts and relationships within the domain.

Ontologies provide a way to map the knowledge and experience that domain experts provide in a computer-readable format [17]. They can form a solid base for recommendations. Ontologies have already been used as a base for decision-support systems for physical activity before. However, the ontologies used were often not developed systematically, were not openly available, or did not follow common guidelines for ontology development, such as the Open Biological and Biomedical Ontology (OBO) Foundry principles [18, 19]. Moreover, available ontologies usually only provide a recommendation for an activity (e.g. “walking”) or an exercise (e.g. “squats”), but do not provide comprehensive planning support, including barriers and coping strategies [18].

### Ontologies for behaviour change

Ontologies are increasingly being recognised as an important tool in the behavioural sciences. A recent consensus report has identified the need for ontologies in the field, focusing on their potential to support communication and classification of knowledge, as well as the integration of knowledge [20]. As such, ontologies can help make research in the behavioural sciences more efficient and reduce research waste. Given that psychological research is currently in a replication crisis [21] and a theory crisis [22] creating clear and unambiguous concepts is particularly crucial.

In a systematic review of ontologies, we identified existing ontologies on physical activity, classified their content and assessed their quality [18]. Though we were able to identify 28 ontologies on physical activity and exercise, only eight of those were openly available, and none of them were considered of sufficiently high quality for reuse. On top of that, the content of ontologies varied strongly, but failed to include relevant information on the context in which physical activity occurred.

In health promotion, ontologies can be applied beyond creating a shared understanding. First, they can improve data integration from different sources, such as electronic health records and wearable devices. For example, the Mining Minds Context Ontology [23] was created in the context of behaviour identification, whereas the Physical ACtivity Ontology [24] was created to support standardising descriptions of physical activities. Second, they can facilitate interoperability and data sharing between systems and stakeholders. For example, the Taxonomy RehAbilitation of Knee conditions Ontology has been created to support knowledge exchange between healthcare professionals and researchers [25], and the FHIR and SSN-based T1D Ontology [26] was developed to connect patients to different service providers with different sources of medical data. Third, ontologies can be used to develop personalised recommendations for individuals based on their specific needs, preferences, and goals [27–29]. This can help to bridge the gap between intention and behaviour, as individuals are more likely to engage in when they feel that the recommendations are tailored to their needs [30].

### Present study

The objective of this project was to create an ontology for a decision-support system for action- and coping plans which (i) promotes general physical activity, (ii) targets Flemish adults who do not meet the World Health Organisation’s guidelines, (iii) is systematically developed and (iv) follows the OBO Foundry principles for ontologies. We refer to this ontology as the COntextualised and Personalised Physical activity and Exercise Recommendations (COPPER) Ontology.

## Methods

In developing the ontology, we followed the steps of ontology engineering, adapted from Pinto and Martins [31], namely specification, conceptualisation, formalisation, implementation and evaluation. Each phase is described in detail below.

**Fig. 1:**
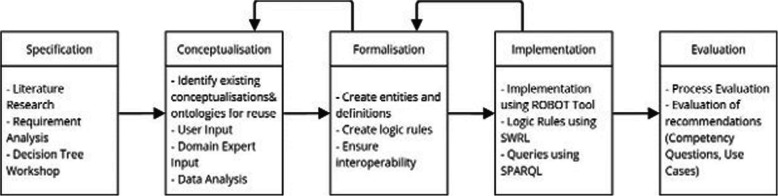
A schematic overview of the design of the copper ontology. Note. The design process consists of five steps, namely the specification, the conceptualisation, the formalisation, implementation and evaluation phase

Figure 1 gives an overview of the design phases of the COPPER ontology, and the activities conducted in each of these phases. It should be noted that these steps did not necessarily occur sequentially. For example, specification and conceptualisation occurred partially in parallel, and implementation was partially carried out before formalisation had finished. The process had an iterative nature, with results of every step being revised after further evaluation.

### Step 1: ontology specification

The goal of ontology specification is to identify the purpose and scope of the ontology. The outcome of this phase were decision trees and personas that informed the future phases.

Ontology specification took place in two main phases. First, the scope and purpose were partially determined through literature research, and built upon previous work of our research group [8, 32]. The scope of the project was to support the planning process by providing personalised recommendations for action and coping plans.

**Fig. 2:**
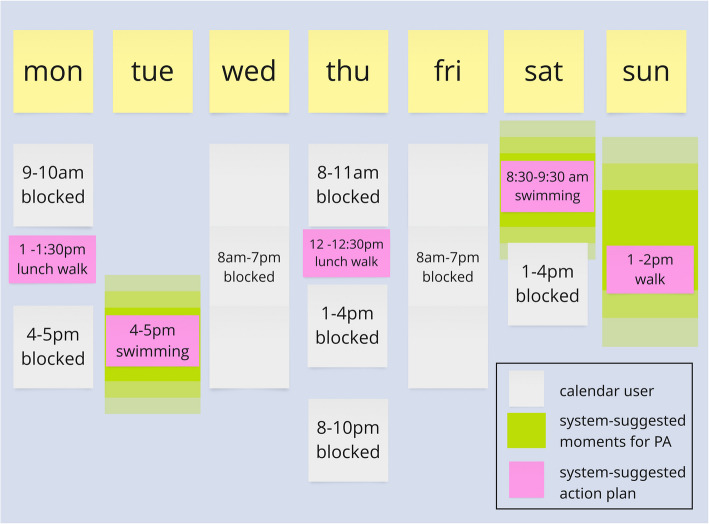
A scenario for the creation of a weekly plan. Note. Based on the user’s calendar (white) and the user’s previous behaviour, the system can suggest moments for the user to plan their physical activity (green). The system then uses this information to make specific suggestions (pink)

Second, together with experts from the domains of Computer Science and the Health and Behavioural Sciences from Ghent University, a requirements analysis was performed to identify the requirements of the decision support system in which the ontology would be used [33]. During the requirement analysis experts were asked to define user stories in the form of “*As a user I want to be able to do... as to achieve....*”. These requirements resulted in the definition of several scenarios and personas that define the interaction of the user with the system. An example of a weekly plan for such a persona is illustrated in Figure 2.

**Fig. 3:**
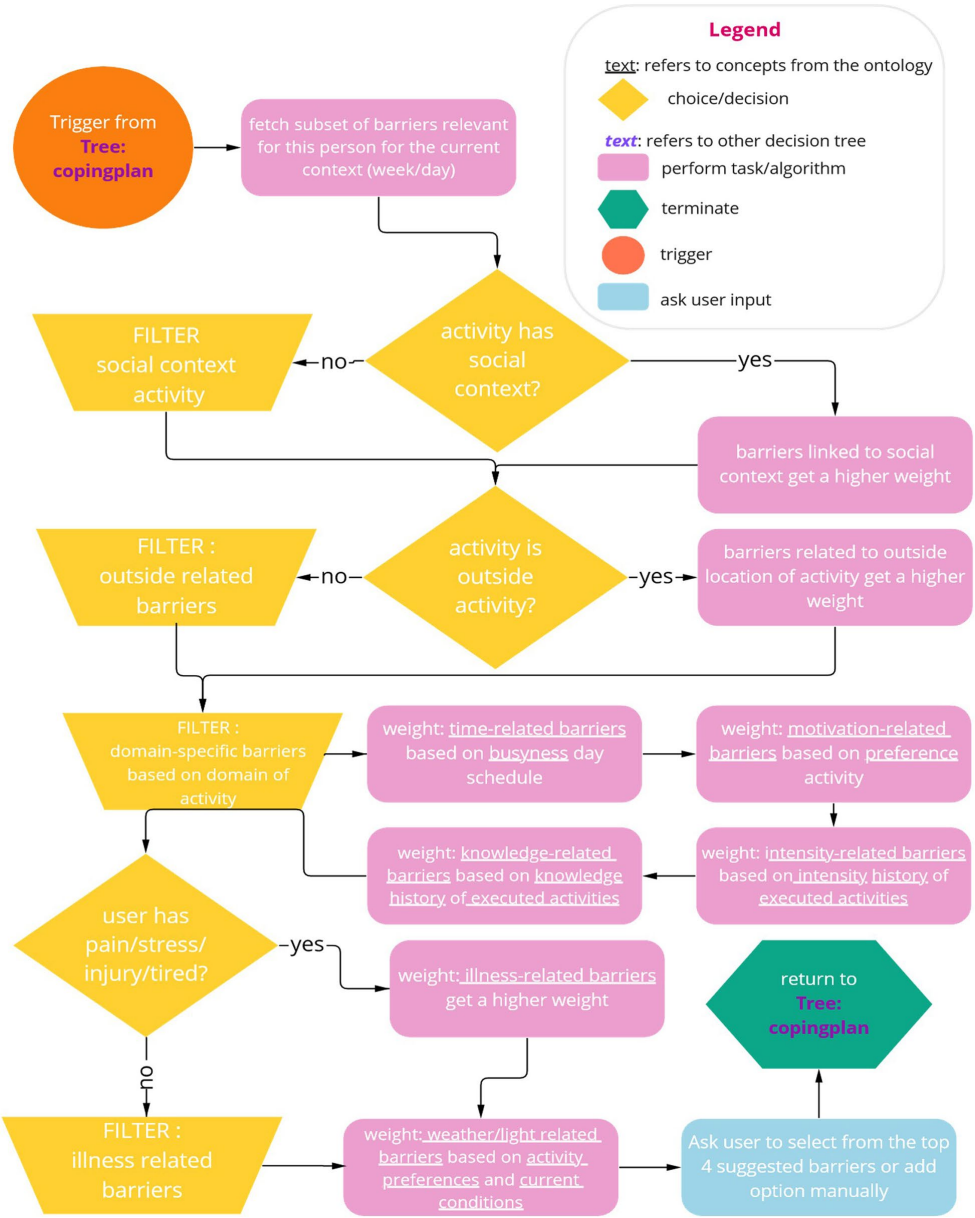
A decision tree for the suggestions of the barriers within a coping plan. Note. A decision tree providing an overview of triggers (orange), choices and decisions within the system (yellow), tasks and algorithms within the system (pink), user input (blue) and termination points (green) when choosing relevant barriers for a given user

Based on these scenarios, decision tree workshops were held with the same group of experts to extract the missing domain knowledge that is needed to inform the ontology [33]. For this, decision trees for a range of activities (e.g. creating your first plan, creating a second plan after reaching one’s goal) were created by two researchers, and feedback for those was sought from the larger group of experts. Figure 3 shows an example of such a decision tree for the suggestions of barriers in a coping plan. The decision trees were used to define missing concepts and relationships of the ontology.

### Step 2: ontology conceptualisation

The goal of ontology conceptualisation is to construct a conceptual model of the ontology. The outcome of this step was a list of classes, including their labels and definitions, as well as relations and properties. We consulted (1) theories and classification systems, (2) researchers in the fields of behavioural change and physical activity, (3) data on physical activity plans, and physical activity itself and (4) end-users.

#### Ontologies and concepts

Reusing existing theories and classification systems as much as possible is a crucial part of ontology development [19]. A summary of the existing ontologies and concepts that were reused or that provided inspiration can be found in Table 1.

**Table 1:** Table summarizing existing models and classification systems that were reused to some extent within the COPPER ontology

Name	Reference	Kind of reuse
Ontologies		
Mechanisms of Action Ontology	[34]	Direct reuse barriers
Behaviour Change Technique Ontology	[35]	Direct reuse coping strategies
Profile Ontology	[36]	Direct reuse profile
Physical Activity Ontology	[24]	Base for list of classes: activities
Other classification systems		
Compendium of physical activities	[37]	Base for list of classes: activities
Domains of physical activities	[38]	Base for list of classes and properties: activities
Self-enactable techniques	[39]	Base for list of classes and definitions: coping strategies
Theoretical models		
Health Action Process Approach	[40]	Base for structure: Plan
Theoretical Domains Framework	[41]	Base for overall structure

We identified the Behaviour Change Intervention Ontology [42], and more specifically the sub-ontologies focusing on profile, behaviour change techniques [35] and mechanisms of action [34], as relevant for re-use. This decision was also made in order to increase interoperability with prominent ontologies within the domain of human behaviour change. However, it should be noted that the Human Behaviour Change Project was still ongoing during the development of the COPPER ontology, with most of their ontologies being published towards the end of our development process. That is why, particularly in early development, we often referred first to other conceptualisations, such as the self-enactable techniques [39].

The use case of the ontology is based on planning interventions that were previously developed within our research groups [8]. Within these interventions, users created action- and coping plans for physical activity, based on the Health Action Process Approach [40]. The following components needed to be represented within the action plan: the activity itself, its duration, starting time, location and whether it was planned to be carried out by oneself or with others. Within the coping plan, potential barriers and their coping strategies needed to be represented.

Concerning the action plans, the main challenge was to represent the activities. We conducted a systematic review of ontologies related to physical activity [18], but found no ontology of sufficient quality to reuse. However, the Physical ACtivity Ontology [24] informed the final list of classes chosen. Further, we considered using the compendium of physical activities [37] as a base for classes. While this was more extensive than necessary for the current specific use-case, we did use the compendium to complement the list we had created. Further, we also considered using the domains of physical activity, namely leisure activities, occupational activities, household activities and active transport [38]. While the domains are not specifically represented within the final ontology, they still influenced the way of thinking throughout development. Some domains, such as household activities, were implemented in the final ontology as a parent class. This was done based on whether the activities within one domain were categorized in that domain (e.g. “cleaning” is only conceptualized within the “household” domain) or whether they could be implemented in multiple domains (e.g. “cycling” can be done for leisure, active transport).

For the barriers within the coping plan, we chose to depart from the mechanisms of action ontology from the Human Behaviour Change Project [34]. This ontology aims to provide a detailed classification of both facilitators and barriers of behaviour, based on domain expert feedback and stakeholder review. For the coping strategies within the coping plan, we considered the behaviour change techniques taxonomy [43] and the compendium of self-enactable techniques [39]. Notably, the behaviour change techniques ontology already included most classes from the self-enactable techniques that were not included in the original behaviour change techniques taxonomy. Within the current project, we reused relevant parts of these ontology, and specified further classes where necessary.

#### User Input

Users, i.e. Flemish adults who do not meet the WHO guidelines for physical activity, were involved in two phases of ontology development. First, users were consulted when determining the scope of the ontology in a group concept mapping study. Second, users were consulted towards the end of the study to map how barriers and coping strategies relate to each other.

*Group Concept Mapping about users’ needs and wishes.* We conducted a group concept mapping study in order to gain insights into users’ needs and wishes for a digital intervention that promotes physical activity. In a first step, we conducted focus groups to collect statements regarding the needs and wishes of users (inactive healthy adults, *n* = 19). After, a new group of users rated and sorted those statements (healthy adults, *n* = 46), allowing for cluster analysis. Six clusters of wishes and needs were identified, with the highest priorities concerning ease of use, self-monitoring, lack of (intrusive) advertisement and technical aspects, such as notifications. A more detailed report on this study can be found elsewhere [44].

The results of this study informed the determination of the scope, and thus the selection of classes. Specifically, it directed the focus of the activities towards everyday activities that are achievable for the target group and did not prioritize the role of social context.

*Questionnaire data connecting barriers and coping strategies.* We conducted a questionnaire study in order to better understand which coping strategies would be relevant for which barriers. For this, we provided participants (healthy adults, *n =* 152) with a scenario and a barrier (e.g. “Imagine you wanted to be active later today. However, you’re worried that you might be tired.”). We then provided a list of possible coping strategies (e.g. “drink a cup of coffee”, “ask a friend to join me”), and asked participants to rate the relevance of each strategy. If participants found strategies to only be useful under certain circumstances, we further asked them to specify which circumstances. This would later allow us to create logic rules connecting barriers and coping strategies. A more detailed report can be found elsewhere [45].

#### Domain experts

Multiple groups of experts were consulted to inform the content of the ontology.

*Continuous and iterative feedback.* First, physical activity and behaviour change experts from Ghent University were consulted throughout the development of the ontology to review the content. Specifically, experts were involved in reviewing the list of classes, definitions and properties of the physical activities, barriers and coping strategies sub-ontologies.

Second, researchers in the domain of Behaviour Change from University College London were consulted concerning the compatibility with existing ontologies, most notably the Behaviour Change Intervention Ontology [36]. For this, instances where classes would be imported from existing ontologies were presented together with the necessary additions for the COPPER ontologies. Appropriateness of the imported classes and relationships with added classes, as well as the choice of label and definition, were discussed.

*Workshops.* Researchers from multiple institutions in Lisbon, Portugal, as well as Ghent University were consulted concerning the relationships between activities and barriers, and barriers and coping strategies, respectively. In order to create those links, a two-hour workshop was organised with researchers from different fields, including behavioural sciences and health sciences.

In the workshop that linked activities and barriers, researchers (*n* = 6) were asked to brainstorm about characteristics of activities, profile and context that would either be necessary for a barrier to occur (e.g. rain can never be a relevant barrier for an activity unless it is outdoors), that would make a barrier more likely (e.g. lack of time is more likely when working full time) or less likely (e.g. rain is less likely during summer). Researchers were asked to first brainstorm about a set of barriers individually for a short time before discussing them in the entire group.

To link barriers and coping strategies, researchers (*n* = 6) were provided with a shortened list of coping strategies, including brief explanations, prior to the workshop. They were then asked to individually reflect about what kinds of barriers could be solved by which coping strategies. This preparation allowed for a more structured and efficient discussion of the links within the workshop, where each researcher was encouraged to also provide input towards coping strategies they did not prepare.

Results from both workshops were summarised and sent to the participants for final feedback.

### Data

Multiple datasets were used. The first dataset contained open text data on action- and coping plans for physical activity. The second dataset contains ecological momentary assessment (EMA) data on physical activities and their contexts. Both kinds of datasets and how they were used to inform the ontology are described in more detail below.

*Text data from action- and coping plans.* The main dataset to inform the ontology contained action- and coping plans concerning physical activity from Flemish psychology students (*n* = 360). Students were instructed to plan each morning for 8 consecutive days, and evaluate their goal progress in the evenings. Resulting data was coded, with the action plan being coded into the variables “activity”, “location”, “social context”, “time” and the coping plan being coded into variables “barrier” and “solution”. A more detailed description of this study can be found elsewhere [46, 47]. The resulting data were aggregated and served as a starting point for the classes within the ontology. A similar dataset^1^ from a sample of patients with fibromyalgia (*n = 34*) were reviewed for additional relevant classes in all categories. For example, “activity-induced pain” was added as a potential barrier based on the fibromyalgia dataset.

*EMA data on physical activity habits.* An event-based user-initiated EMA study was conducted to collect data on physical activity and its context. Participants (*n = 52*) wore an accelerometer for 14 days, and filled in questionnaires every morning, every evening, and every time they moved for at least five minutes. In addition, participants filled in an intake questionnaire prior to the beginning of the EMA period and took part in a semi-structured interview after completion. This study is explained in more detail elsewhere [48].

The resulting dataset contained (1) baseline information on the participant, including sociodemographic information, information on their health and social cognitive determinants of physical activity, (2) daily participant information, including affective and physical states and dynamical measures of social cognitive determinants, (3) information on each activity, including information on activity type, intensity, and social and physical context. Some further questions concerned behavioural cognitions of the participant regarding the current activity.

The data from this study was used to inform connections between different classes within the ontology - most importantly, it allowed connecting different context and profile characteristics to specific activities.

#### Building the conceptual model

At this stage, information was triangulated in different formats, including spreadsheets to define classes, definitions and properties (see also ontology formalisation), matrices to conceptualise rules (e.g. to match barriers to coping strategies), and diagrams to provide higher-level overviews of the structure (see e.g. figure 5). These were created ad hoc according to in-the-moment requirements and needs.

The information from the various sources was triangulated to build a conceptual model in different ways. First, where work happened consecutively, input from previous work was used to inform future work. For example, the text data from action- and coping plans informed the available barriers and coping strategies in both the user survey and the domain expert workshops later. Second, information from multiple sources was jointly considered when creating the classes and rules. We opted to be as inclusive as possible when it came to creating classes to capture as many important concepts as possible. For example, we included barriers and coping strategies that were not deemed as highly relevant by experts but nevertheless, were frequently reported in in the action- and coping plan data, except when considered harmful. As ontological rules represent restrictions, we were conservative when it came to creating rules in order not to exclude potentially relevant connections. For example, while experts judged some coping strategies not to be highly relevant for certain barriers end-users considered them relevant in the online task. As such, we did not exclude the coping strategies as potentially relevant for the barriers within our rule set. Third, the conceptual model was reviewed by domain experts from Ghent University, Belgium, before formalization.

### Step 3: ontology formalisation

The goal of ontology formalisation is to translate the conceptual model into a formal model. As such, it is strongly tied to both conceptualisation and implementation, serving as a bridge between the two.

For formalisation, structured spreadsheets were created for each lower-level ontology by SC. In these spreadsheets, names of relevant concepts, their definitions and relationships to other constructs were noted, and iteratively adapted from the beginning of conceptualisation up until the end of the implementation based on new information and researcher feedback. Similarly, in order to define logic rules, rules were created in a spreadsheet, specifying involved concepts and how they relate to each other. Spreadsheets created throughout formalisation were human readable and not yet machine-readable. However, in later stages of development, spreadsheets that were used as input for the ROBOT tool (s. subsection implementation of entities) were simultaneously used for formalisation. The spreadsheets are openly available on osf [49]

### Step 4: ontology implementation

The goal of ontology implementation is to translate the formal model into a computer-readable model. For this, we used the Web Ontology Language (OWL) [50] using Protégé.

#### Implementation of entities

**Fig. 4:**
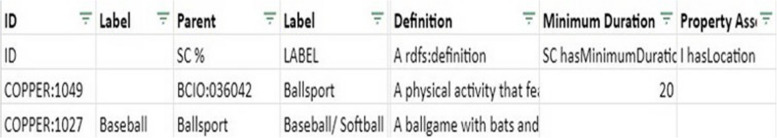
A snippet of the CSV file for the Activities ontology. Using the ROBOT tool, the csv files are translated to OWL syntax. Note. The spreadsheet contains a unique identifier (ID), label, parent (can be indicated using the ID or the label), definition, minimum duration (a data property) and location (an object property)

The ROBOT tool [51] from OBO was used to implement the classes and properties of the ontology. This tool facilitates automating repetitive tasks. Classes and properties for each lower-level ontology were defined in a separate CSV file, as shown in Figure 4. Using the ROBOT tool, these CSV files can be transformed into OWL files and merged into a single ontology. This simplified the repetitive task of adding a large number of entities to the ontology. The ROBOT tool was implemented within this project by SC. Protégé version 5.6.3 was used to check the consistency of the ontology by running the Pellet reasoner.

Each of the newly defined entities in the COPPER ontology received an ID according to a predefined structure based on the lower-level ontology it belongs to, as shown in Table 2.

**Table 2:** Newly defined entities in the COPPER ontology receive an ID based on the lower-level ontology they belong to

Ontology	ID set
Planning	COPPER:0000 - COPPER:0999
Activities	COPPER:1000 - COPPER:1999
Context	COPPER:2000 - COPPER:2999
Barriers	COPPER:3000 - COPPER:3999
Coping Strategy	COPPER:4000 - COPPER:4999
Profile	COPPER:5000 - COPPER:5999

#### Implementation of logic rules

Logic rules were implemented using a Semantic Web Rule Language (SWRL, [52]) by MB, and reviewed by SC and FDB. Each rule was tested on a use-case upon implementation. In order to document the rules, human readable descriptions of each rule were noted in a spreadsheet. Additional notes were taken for the code needed to implement the rule, which step in evaluation it is relevant for (e.g. “barrier recommendation”) and which lower-level ontology it is based on (e.g. “profile”). Each rule received an individual label containing the step (e.g. rules relevant for activity recommendations start with 1), an ongoing number, and a letter if multiple rules were related to each other. An overview of these rules can be found on osf [49].

### Step 5: ontology evaluation

We carried out two types of evaluation. First, the process evaluation concerns principles of ontology development throughout the development process. Second, the content evaluation concerns whether the ontology, when applied, provides suitable recommendations.

#### Process evaluation: commitment to OBO foundry principles

In order to evaluate the development and maintenance of the ontology, a checklist was created based on the OBO foundry principles for good practice. These principles are the ones used to evaluate ontologies’ suitability for the OBO Foundry [53], and have been the base for evaluating ontologies in reviews [18, 54]. They concern the development process, the ontology itself, the maintenance and documentation. The criteria were slightly adapted from [18], and can be found in full in Supplement 1.

#### Content evaluation

The content of the ontology was evaluated in two ways.

First, a range of competency questions was created by MB and SC, and reviewed by all authors. Competency questions are frequently used in ontology development in order to evaluate the scope of the ontology [55]. In short, they are questions that the ontology needs to be able to answer. They were based on the requirement analysis carried out during ontology specification. Competency questions were structured by the order in which recommendations are provided, starting with activity recommendations (e.g. the ontology must be able to take household composition into account when recommending activities), then context recommendations (e.g. the ontology must know which locations are appropriate for which activity), then barrier recommendations (e.g. the ontology must take sociodemographic factors (age, gender, socio-economic status) into account when recommending potential barriers) and last coping strategy recommendations (e.g. the ontology must know which coping strategies are relevant for which barriers). A full list of competency questions and use-cases can be found in Table 5.

Second, a series of use cases was created in order to test the ontology. Each use-case consisted of a simple profile (gender, age, occupation, living situation), including fitness level and activity-related preferences. Each use case was implemented in the ontology as instances, and their recommendations were retrieved using SPARQL queries. For each step (activity, context, barrier, coping strategy), all relevant recommendations were queried and a choice was made before advancing to the next step. Recommendations were evaluated based on the competency questions by MB, and reviewed by all authors.

## Results

### Ontology description

The resulting ontology focuses on day-level physical activity and exercise action and coping plan recommendations for physical activity and exercise in inactive Flemish adults. The goal of the ontology is to reduce the risks of physical inactivity.Within the ontology, we follow the WHO’s definition of physical activity as any bodily movement produced by skeletal muscles that requires energy expenditure, referring to all movement including during leisure time, for transport to get to and from places, or as part of a person’s work or domestic activities [56]. Some of the physical activities we include in the ontology can be considered exercise, i.e. planned, structured and purposeful physical activity [38]. This is the case for e.g. treadmill running or yoga, but not necessarily for other activities e.g. household activities.

**Fig. 5:**
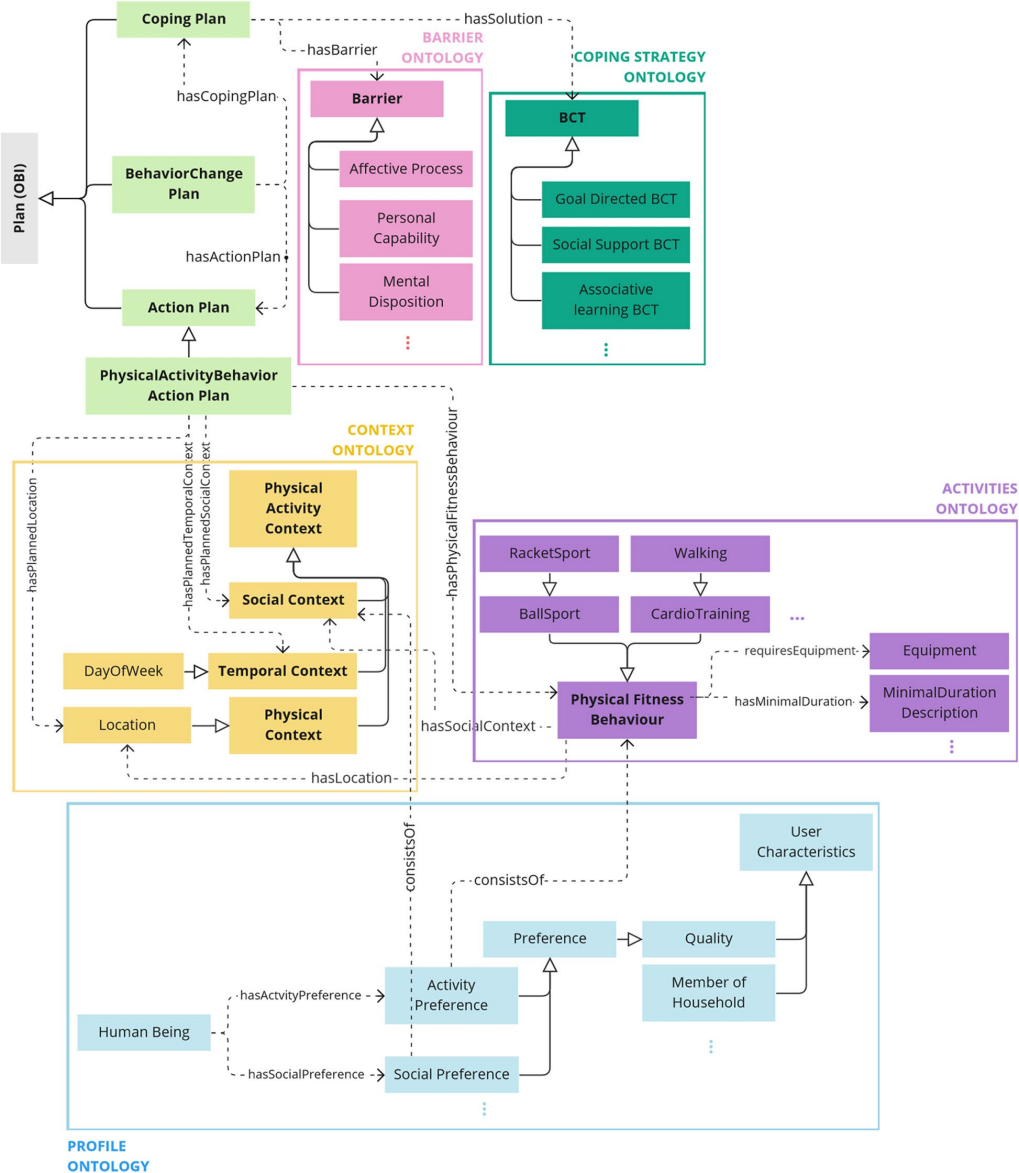
A high-level overview of the copper ontology with the upper-level ontology and some of its relationships to the lower-level ontologies. Note. The ontology contains lower-level ontologies, examples of classes within them, and key relationships between and within the lower-level ontologies

The COPPER ontology consists of multiple lower level ontologies, focusing on profile, planning, activity, context, barriers and coping strategies, respectively. Please find an overview of the descriptions characteristics of each lower-level ontology, as well as the full ontology in Table 3. Figure 5 shows a high-level overview of the upper ontology structure with some of the concepts in the lower-level ontologies.

**Table 3:** Counts of classes, instances and properties for each lower-level ontology and the total COPPER ontology at the moment of submission

Ontology	Description	Classes	Instances	Object Properties	Data Properties
Profile	This ontology describes individual-level characteristics of potential users, such as age, gender, or occupation.	22	45	28	2
Planning	This ontology provides structures for the remaining lower-level ontologies. It distinguishes between action and coping plans and determines the relationships between different kinds of plans, i.e. the fact that one action plan can have multiple coping plans, but each coping plan only refers to one action plan. Action plans consist of information about the activity and context, while coping plans consist of information about the barrier and coping strategy.	14	0	26	1
Activity	This ontology describes different activities that can be recommended, as well as their properties.	67	132	10	5
Context	This ontology describes the temporal, physical and social context an activity can have.	16	14	0	0
Barrier	This ontology describes possible barriers that can occur when people are attempting to be physically active. In creating this ontology, we focused on barriers that can be overcome by an individual on a day-to-day basis. We thus did not include structural or societal barriers, such as walkability	90	50	0	2
Coping Strategy	This ontology describes the coping strategies that people can use to either prevent a barrier from occurring, or to deal with a barrier once it has occurred. We have focused on coping strategies that can be implemented in the short term (i.e. doing a lighter exercise when it is to hard) rather than long term (i.e. gaining body strength in order to be able to do a certain exercise).	96	63	0	0
COPPER		288	312	64	9

### Process evaluation: commitment to OBO principles

The ontology development process was evaluated using the OBO principles of ontology development. All principles were followed by the researchers, as demonstrated in Table 4. It should be noted that while the infrastructure is already provided for the last three criteria, they were not yet relevant for COPPER. The COPPER ontology was found to be logically consistent.

**Table 4:** Overview of application of obo foundry principles of ontology development

Principle	Application
Open	The COPPER ontology is openly available via GitHuba and OSFb, and will be submitted to the BSSO foundry.
Common Formal Language	The COPPER ontology is available in an owl file using the RDF-XML syntax.
Unique URI	Each class and property in COPPER has a unique uniform resource identifier.
Versioning	Version control is available via GitHub and OSF, including comments describing the changes made in each version.
Textual Definitions	Textual definitions following guidelines for ontological definitions [50] were created for all classes.
Naming Conventions	COPPER uses clear naming conventions.
Documentation	The current paper serves as documentation for the development and structure of the COPPER ontology.
Locus of Authority	We provided a clear point of contact in GitHub, though long-term point of contact still needs to be established.
Reuse	We have documented the reuse of both ontological and non-ontological resources throughout this paper. Further, ontological reuse can be recognized within the ontology by the original IDs, and reuse of non-ontological sources is noted in a comment for each class
Documented Plurality of Users	We provide space for an overview of projects using the COPPER ontology on OSF and GitHub. Documenting usage of the COPPER ontology that we were made aware of is part of the maintenance plan.
Maintenance	The ontology is planned to be maintained and updated in the context of the projects it will be used in.
Responsiveness	An issue tracker was implemented via GitHub.

^a^https://github.com/EBehaviourChange-COPPER/ontology

^b^https://OSF.io/rm5pg/

### Content evaluation

Content evaluation was done in two ways. First, competency questions were created. Second, multiple use cases were implemented into the ontology to test whether logic rules were implemented correctly, and the provided recommendations followed them.

#### Competency questions

Competency questions were created and answered in a step-by-step fashion, following the usual steps of action and coping planning. In its current stage, only logic rules that decide over which recommendations are relevant or not relevant in a binary fashion are implemented. That means that no probabilistic rules are implemented at this stage. In the following, we will discuss which of the developed competency questions are already answered by the current version of the ontology, and which will be addressed in future work.

A total of 22 competency questions were developed in the context of ontology development and evaluation. In its current state, the COPPER ontology is able to fully answer 12 of these competency questions, partially answer four of them, and not yet answer six of them. Where COPPER is unable to answer (parts of) competency questions, this is usually due to one of two reasons. First, at this stage, only logic rules have been implemented in COPPER. However, many relevant relationships that could be defined for relevant recommendations are probabilistic. These rules will be covered in future work. Second, some entities that are required to create rules related to weather, sunlight and temporal context are highly dependent on the technological implementation of an intervention using COPPER. This was particularly true for concepts that would be derived from sensor data. Hence, creating these rules was not within scope for the development of the ontology. Table 5 displays an overview of all competency questions, whether they were answered within the current version of the COPPER ontology, and if so, what was implemented and in which logic rules, and what future work is still needed. A more detailed, textual overview of how the different competency questions are implemented and what future work is planned can be found on OSF [49].

**Table 5:** Overview of current status of all competency questions

**Competency Question**	**Answered?**	**Implementation**		**Future work**	
		**Specification**	**Rel. Logic Rules**	**Prob. Rules**	**Techn. Impl.**
**Recommending a suitable activity given a certain profile**					
CQ1.1 The ontology must be able to take preferences and aversions into account when recommending activities.	Yes	Social and duration preferences, activity aversions	1 - 1 A – 1 - 1P		
CQ1.2 The ontology must be able to take sociodemographic factors (age, gender, socio-economic status, employment status, household composition) into account when recommending activities.	Partially	No expensive activities for people with below average income	1 - 1 A – 1 - 1P	X	
CQ1.3 The ontology must be able to take prior experience with certain activities into account when recommending activities.	No		1 - 1 A – 1 - 1P		X
CQ1.4 The ontology must be able to take the current fitness level into account when recommending activities.	Yes	No high intensity activities for people with low fitness	1 - 1 A – 1 - 1P		
**Recommending a suitable context given a certain profile and activity**					
CQ2.1 The ontology must know which locations are appropriate for which activity.	Yes	Possible locations and space mapped per activity	Implemented via object properties		
CQ2.2 The ontology must know which social context is appropriate for which activity.	Yes	Possible social context mapped per activity	Implemented via object properties		
CQ2.3 The ontology must know how long different activities take.	Yes	Minimum duration mapped per activity	Implemented via object properties		
CQ2.4 The ontology must take the weather into account when recommending activities.	No				X
CQ2.5 The ontology must take daylight into account when recommending activities.	No				X
**Recommending suitable barriers given a certain profile, activity and context**					
CQ3.1 The ontology must take sociodemographic factors (age, gender, SES) into account when recommending potential barriers.	Partially	Gender-specific barrier (e.g. menstrual pain)	3–4	X	
CQ3.1a The ontology must take other responsibilities (work, household, childcare) into account when recommending barriers.	No			X	
CQ3.2 The ontology must take characteristics of the planned activity into account when recommending potential barriers.	Yes	Barriers based on required experience, material	3–5, 3–7		
CQ3.3 The ontology must take location characteristics into account when recommending potential barriers.	Yes	Barriers based on activity type and space (outside/inside)	3 - 1, 3 - 2, 3 - 3		
CQ3.4 The ontology must take social context characteristics into account when recommending potential barriers.	Yes	Barriers based on social context and possible social context	3 - 6 A, 3 - 6B		
CQ3.5 The ontology must take temporal context characteristics into account when recommending potential barriers.	No	Barriers based on activity duration		X	X
**Recommending suitable coping strategies provided profile, activity, context and barriers**					
CQ4.1 The ontology must know which coping strategies are relevant for which sociodemographic groups.	No	Strategy involving pets only if individual has pets	4–23	X	
CQ4.1a The ontology must be able to take other responsibilities (work, household, childcare) into account when recommending barriers.	No			X	
CQ4.2 The ontology must be able to take characteristics of the planned activity into account when recommending potential coping strategies.	Yes	Based on possible social context, equipped material, intensity	4 - 19 A-C, 4 - 20 A-D, 4 - 22 A-B		
CQ4.3 The ontology must be able to take location characteristics into account when recommending potential coping strategies.	Yes	Some coping strategies only for indoor or only for outdoor activities	4–16, 4–17, 4–18		
CQ4.4 The ontology must be able to take social context characteristics into account when recommending potential coping strategies.	Yes	Some coping strategies only for solo or only for group activities	4–16, 4–17, 4–18		
CQ4.5 The ontology must be able to take temporal context characteristics into account when recommending potential coping strategies.	Partially	Based on planned duration	4 - 21 A-D		X
CQ4.6 The ontology must know which coping strategies are relevant for which barriers.	Yes	One on one mapping of barriers to coping strategies	4 - 2– 4 - 15B		

#### Demonstration: Use Case Petra

We have tested the ontology using five use cases. For demonstration purposes, we will elaborate one of the use cases. The remaining use cases, as well as recommendations for each step and a short elaboration why certain elements are recommended or not recommended, can be found on OSF in tabular form [49], and in textual form for each use case in Supplementary File 1.

Use case 1 is a 44 year-old female user called “Petra”. She is a married stay-at home parent, with an average household income. Her highest diploma is a PhD. She has a pet dog. She has a preference for activities that can be carried out alone, and are of short duration.

*Step 1: Activity Choice.* Based on the profile, this user should be suggested any activities that can be carried out by oneself, and have a minimum duration of less than 20 minutes. No restrictions are given by the household income or the fitness level. The provided recommendations meet these criteria.

A total of 22 activities meet these criteria and are provided as recommendations. Out of those, “Outdoor Walking” is chosen as an activity for the next steps of the evaluation.

*Step 2: Context Choice.* Based on the activity, this user should be suggested locations and spaces that are possible for walking. Based on the profile and activity, this user should only be suggested carrying out this activity by herself. A minimum recommendation for duration is provided based on the activity.

The person is recommended to carry out this activity outside and by herself, for a minimum duration of 5 minutes. Four different locations are suggested. All recommendations meet the criteria. Within this use case, Petra chooses to walk in a park by herself for 20 minutes.

*Step 3: Barrier Choice*. Based on the context, barriers that are only relevant to outside activities should be provided. Barriers that concern other spaces, such as facilities, should not be provided. Based on the activity, no barriers concerning required expertise, required social context or required equipment should be suggested. Based on the profile, female gender specific barriers should be provided.

A total of 40 barriers meet these criteria and are provided as recommendations. Within this use case, lack of motivation is chosen as a barrier.

*Step 4: Coping Strategy Choice.* Based on the chosen barrier, coping strategies concerning motivation should be suggested. Based on the chosen context and barrier, planning inclusion of enjoyment should be suggested. Solutions specific to indoor contexts should not be suggested. Based on the chosen activity, integrating taking pictures and goal integration can be suggested. Coping strategies specific for group activities, long activities, or high intensity activities should not be included. Based on the profile, coping strategies that include a pet can be included.

Thirty-two coping strategies meet these criteria and are suggested. Within this use case, installing a reward for completing the activity was chosen as a coping strategy.

## Discussion

The goal of the present project was to develop an ontology for recommendations for physical activity action and coping plans. We followed the OBO Foundry principles for ontology development. The resulting COPPER ontology contains 288 classes, 64 object properties and 9 data properties. The recommendations based on COPPER were found to be relevant using competency questions and use cases. COPPER aims to promote physical activity for inactive Flemish adults, but is likely to translate well to other groups, e.g. individuals from other Western countries or clinical groups, by complementing it with additional relevant activities, barriers and coping strategies.

Ontologies have been used previously to provide recommendations for physical exercises or activities [24, 25, 28]. However, the ontologies used previously did not follow the OBO Foundry principles, with many of them not being openly available and lacking definitions of their classes [18]. This made them largely not reusable. The COPPER ontology is the first openly available, reusable ontology for physical activity action and coping plan recommendations, and builds strongly on existing ontologies within the domain of behavioural sciences. It is also the first ontology that comprehensively includes action and coping plans, as opposed to just activities.

Building upon this initial version of the ontology, some further steps need to take place in order to make COPPER useful in interventions: first, the ontology needs to be implemented into a wider decision support system. Within this system, probabilistic rules will be integrated, and technological decisions will be made that allow us to create additional rules based on context variables. This will allow us to answer the remaining competency questions, and thus improve the recommendations provided by COPPER further. Second, the recommendations provided by the ontology have not yet been evaluated beyond use cases. Future work could do this in several ways: First, a study could investigate whether domain experts rate the recommendations provided by an ontology-based decision-support system as more relevant than random recommendations using a simplified user interface. Second, a planning-based digital health intervention could be developed, using the ontology-based decision-support system as its base. The intervention could then be evaluated by end users, and compared to one that uses user-created plans, or random suggestions. Last, recommendations based solely on the ontology will always be relatively static, and do not sufficiently take dynamic individual characteristics, such as previous engagement with the recommendations, into account. However, two individuals might have approximately the same profile (e.g. young woman working an office job), but vastly different habits. The COPPER ontology forms a strong base for a decision-support system, and should be accompanied by more flexible algorithms, such as machine learning techniques, taking a user’s activity history and choices in plan creation should be taken into account when creating recommendations. However, it should be noted that the goal of the ontology will never be to provide one perfect recommendation, and rather to provide a selection of suitable suggestions, leaving final agency over the plan to the end-user.

### Challenges and opportunities for ontologies in health psychology

Ontologies are a promising avenue for creating transparent recommendations for physical activity action and coping plans. Being able to not only represent a vast number of entities and their respective characteristics, but also connect them with specific relationships allows us to map existing expertise. Based on logic rules, reasoners can then infer which possible plans are relevant under which circumstances. However, developing and using ontologies also brings some challenges.

A first challenge in ontology development within behavioural sciences is that the knowledge we like to map often does not exist in sufficient level of detail. In the case of the COPPER ontology, very little research was available concerning which type of activity is suitable for whom under which circumstances. This means that we have to more strongly rely on (correlational) data and expert judgement to create rules within the ontology. These rules thus need to be evaluated in experimental designs.

Beyond providing clear recommendations, ontologies have the potential to improve health psychological research by enforcing a strict vocabulary which should lead to improved clarity and decrease the risk of ambiguity [17]. This is both a challenge and an opportunity: as there are currently limited guidelines for creating ontologies within behavioural science [57, 58], arriving at adequate labels and definitions can be exceedingly challenging. Ontology engineers usually depart from an existing clear and unambiguous understanding of the knowledge that should be modelled within the ontology – something that is rarely, if ever, the case in psychological research [58].

Working on definitions for and links between classes also forces researchers to think about the concepts they work with and their relationship in a more detailed way, possibly revealing gaps or contradiction in existing research. There are currently initiatives to create uniquely identifiable constructs with unambiguous definitions for behavioural sciences, even outside of creating ontologies [59]. In creating these constructs, behavioural sciences must strive to find a balance between attempting to establish one shared vocabulary - as is the aim of e.g. the Human Behaviour Change Project [42] - or ontological pluralism, which allows for different perspectives to co-exist, and even be connected [20]. While the former can accelerate research by facilitating communication and aggregation of knowledge, it can also hinder or even halt progress by limiting the dominant discourse to one main perspective. Either way, no matter where one’s stance is in this debate, we must strive towards uniquely identifiable constructs that are well-defined.

Among the main challenges concerning the inclusion of ontologies in health psychology research are the high up-front investment when it comes to both time and resources and the relative lack of expertise within the discipline when it comes to ontology development. As projects often aim to both develop and evaluate digital systems to promote behaviour change in a limited amount of time, spending multiple years on ontology development does not always seem feasible, and is hard to realize within the current funding structure. We believe that this issue needs to be tackled on a systemic level, as the reusability and adaptability of ontologies can make ontologies a worthwhile investment. Especially within one health behaviour, multiple interventions targeting different groups can be derived from the same ontology given some (relatively minor) adaptations. However, there are limits to reuse and adaptations. Most importantly, considerable expertise with ontologies is required in order to reuse or adapt them at all. As ontologies are not widespread in behavioural sciences, this is a severe limitation. As such, ontologies will only be able to reach their full potential once they are more widely used in the research community.

### Limitations

The current study has some limitations.

First, while we have involved input from end-users within the specification and conceptualization phases, their involvement could have been more extensive. Similarly, mostly researchers were involved as domain experts, while the perspective of other stakeholders, such as medical professionals or coaches, could have been a valuable addition. In future work, it should be considered to include a broader range of stakeholders, including end-users and medical professionals, for example as part of an advisory group.

Second, some of the data used to inform the ontology was not collected specifically within the target group of insufficiently active healthy Flemish adults, but in convenience samples, such as student samples. Moreover, some of the data that informed the ontology was collected while government measures were active due to the COVID- 19 pandemic. As the selection and structure of the classes within the ontology was guided by these data, this might have resulted in missing classes or missing links between classes. Later on, our ontology may be further expanded by information residing in publicly available datasets.

Third, the ontology currently only supports users with the goal of reducing their inactivity in a broad sense. It does not currently support more targeted physical activity, e.g. to increase muscle strength, to lose weight, or to improve cardiovascular fitness. While it includes some activities that can be suitable for these goals (e.g. strength training, swimming, running), it lacks detail to support targeted training, nor does it contain a structure to map activities to different goals. However adapting the COPPER ontology to more specific goals is possible.

Last, evaluation of the ontology was limited to process evaluation and use cases. In the future, more thorough evaluation of the recommendations provided by the ontology needs to be carried out. This could be done in a longitudinal design, comparing action and coping plans created based on COPPER with those created with no support or generic support in terms of acceptability as well as plan enactment.

## Conclusions

Physical activity promotion has received increasing attention in recent years, and planning interventions are commonly used to increase physical activity. Providing personalised and context-aware recommendations for action and coping plans can decrease user-burden and increase plan quality. This can be done using black-box data-based approaches [11], but those approaches are not transparent and can easily result in inappropriate recommendations. The COPPER ontology aims to provide personalised recommendations for physical activities in an explainable way. This was achieved by mapping knowledge from existing conceptualisations, end-users, domain experts and data. COPPER contains 288 classes, 64 object properties and 9 data properties, and is developed to be interoperable with other prominent ontologies within the field of behavioural sciences, most notably the Behaviour Change Intervention Ontology. It is structurally and logically consistent, and the resulting recommendations proved relevant in use case evaluation and answered a majority of competency questions, but further evaluation is needed. COPPER can be used as a base for decision-support systems that provide recommendations for action- and coping plans.

## Supplementary Information

Supplementary Material 1.

Supplementary Material 2.

## Data Availability

All materials, as well as links to previous studies and their respective repositories, can be found at https://doi.org/ng6g.

## Abbreviations

COPPER: COntextualised and Personalised Physical activity and Exercise Recommendations
EMA: Ecological Momentary Assessment
OBO: Open Biological and Biomedical Ontology
OWL: Ontology Web Language
PA: Physical activity
ROBOT: Robot is an OBO Tool
SWRL: Semantic Web Rule Language
WHO: World Health Organisation

## Footnotes

1 See https://lib.ugent.be/catalog/rug01:003012045 for an analysis of a subset of this dataset, and more information about data collection.
