# Supplementary material for: Development and evaluation of the COntextualised and Personalised Physical activity and Exercise Recommendations (COPPER) Ontology
Source: Int J Behav Nutr Phys Act. 2025 May 5;22:52. doi: 10.1186/s12966-025-01744-5 (PMC12054263; doi:10.1186/s12966-025-01744-5)
Supplement: Supplementary file 1 — Supplementary Material 1. [file 12966_2025_1744_MOESM1_ESM.docx]

# Supplement 1: Criteria for process evaluation of the COPPER ontology

1. Open: The ontology should be openly available on the internet. Being available upon request was not sufficient.
2. Common Formal Language: The ontology should be available in an OWL file using the RDF-XML syntax.
3. Unique URI: This criterion is met if each class and property had a unique uniform resource identifier (URI), which is a unique characters sequence that distinguishes one resource from another
4. Versioning: Versions should be labelled clearly, including their date of publication and the changes made.
5. Textual Definitions: An ontology should have definitions for the majority of its classes, in particular for top level terms.
6. Naming Conventions: An ontology should have clear naming conventions. This criterion was met if names were unique and intelligible to the coding team MB and SC.
7. Documentation: Significant documentation should be available, e.g. in a published paper describing the ontology, websites, or in manuals for developers and users.
8. Locus of Authority: Contact details (at least name and email address) of a person must be provided. A corresponding author of a publication is sufficient, but the email address needed to be valid (i.e. not return an error notification).
9. Reuse: Ontology developers should reuse ontological or nonontological resources during development. This criterion is met if there was clear documentation that content was imported from other ontological or nonontological resources.
10. Documented Plurality of Users: Usage of the ontology by multiple independent people or organizations must be documented in a freely available online document. This information must be provided by the ontology developers, not those using the ontology.
11. Maintenance: Ontology providers should have a plan for maintaining the ontology, and provide this information in the documentation. Maintenance must take place regularly.
12. Responsiveness: Ontology developers must provide channels for community participation and be responsive to requests. This criterion is met if developers had set up a way to track community requests and suggestions (e.g. an issue tracker).
