# Supplementary material for: Development and evaluation of the COntextualised and Personalised Physical activity and Exercise Recommendations (COPPER) Ontology
Source: Int J Behav Nutr Phys Act. 2025 May 5;22:52. doi: 10.1186/s12966-025-01744-5 (PMC12054263; doi:10.1186/s12966-025-01744-5)
Supplement: Supplementary file 2 — Supplementary Material 2. [file 12966_2025_1744_MOESM2_ESM.docx]

# Supplementary File 2: Additional Use Cases

## Case evaluation 2 Christa

Use case 2 is a 57 year-old female user called “Christa”. She is widowed, and does not work for health reasons. She has a low household income. Her highest diploma is at bachelor level. She has no social preference, but prefers short activities.

***Step 1: Activity Choice.*** Based on the profile, this user should be suggested any activities that have a minimum duration of less than 20 minutes. No restrictions are given by social preferences. Due to the low household income, no expensive activities or activities that require a membership should be recommended. Due to the fitness level, no high intensity recommendations should be provided.

A total of 17 activities meet these criteria and are provided as recommendations. Out of those, “Pilates” is chosen as an activity within this use case.

***Step 2: Context Choice.*** Based on the activity, this user should be suggested locations and spaces that are possible for pilates. This includes both indoor and outdoor locations. A minimum recommendation for duration is provided based on the activity. Any social context is possible for pilates. The provided recommendations meet these criteria.

Within this use case, Christa chooses to do pilates by herself at home for 10 minutes.

***Step 3: Barrier Choice***. Based on the context, barriers that are only relevant to outside activities should not be provided. Barriers that concern other spaces, such as facilities, should not be provided. Based on the activity, no barriers concerning required expertise, required social context or required equipment should be suggested. Based on the profile, female gender specific barriers should be provided.

Thirty barriers meet these criteria and are provided as recommendations. Within this use case, pain is chosen as a barrier.

***Step 4: Coping Strategy Choice.*** Based on the chosen barrier, coping strategies concerning motivation or pain should be suggested. Based on the chosen context and barrier, planning inclusion of enjoyment and asking others to join should be suggested. Solutions specific to indoor contexts should be suggested. Based on the chosen activity, integrating taking pictures and goal integration cannot be suggested. Coping strategies specific for group activities, long activities, or high intensity activities should not be included. Based on the profile, coping strategies that include a pet cannot be included.

Thirty-three coping strategies matched these criteria. Within this use case, non-pharmaceutical pain management is chosen as a coping strategy.

## Case evaluation 3 Tobias

Use case 3 is a 34 year-old male user called “Tobias”. He is single, works full-time and has an average household income. He has no pets. He is educated at master level. He prefers activities he can carry out with someone else, and prefers long activities. He has a high fitness level.

***Step 1: Activity Choice.*** Based on the profile, this user should be suggested any activities that can be carried out in group. No restrictions are given by the household income, duration preference or fitness level.

44 activities meet these criteria and are provided as recommendations. Within this use case, “Padel” is chosen as an activity.

***Step 2: Context Choice.*** Based on the activity, a sports facility is recommended as the only possible location. The activity can be performed indoor and outdoor, either with one additional person or in group. A minimum duration of 20 minutes is recommended.

Within this use case, an outdoor Exercise Facility is chosen, the activity is planned with one other person and for 60 minutes total.

***Step 3: Barrier Choice.*** Based on the context, barriers that are only relevant to outside activities should be provided. Barriers that concern sport facilities should be provided. Based on the activity, barriers concerning required expertise, required social context or required equipment should be suggested. Based on the profile, female-gender specific barriers should not be provided.

A total of 48 barriers meet these criteria and are provided as recommendations. Within this use case, feeling hungry is chosen as a barrier.

***Step 4: Coping Strategy Choice.*** Based on the chosen barrier, coping strategies concerning motivation should be suggested.

Based on the chosen context and barrier, planning inclusion of enjoyment and asking others to join, integrating taking pictures and goal integration should not be suggested. Solutions specific to indoor contexts should not be suggested. Specific coping strategies for hunger should be suggested. Coping strategies only relevant for group activities, long activities, high intensity activities or activities that require equipment should be included.

34 coping strategies meet these criteria and are provided as recommendations. Within this use case, taking breaks whenever needed is chosen as a coping strategy.

## Case evaluation 4 Andreas

Use case 4 is a 28 year-old male user called “Andreas”. He is in a committed long-term relationship, works full-time and has a low household income. He has no pets. He is educated at bachelor level. He has no preferences concerning the social context or duration of his activities. He has an intermediate fitness level

***Step 1: Activity Choice.*** Based on the profile, this user should be suggested any activities that is affordable. No restrictions are given by the fitness level, duration preference, social preference or fitness level.

38 activities meet these criteria and are provided as recommendations. Within this use case, “Badminton” is chosen as an activity.

**Step 2: Context Choice.** Based on the activity, a sports facility is recommended as the only possible location. The activity can only be performed indoors, either with one additional person or in group. A minimum duration of 20 minutes is recommended.

Within this use case, an indoor Exercise Facility is chosen, the activity is planned in group and for 90 minutes total.

**Step 3: Barrier Choice.** Based on the context, barriers that are only relevant to outside activities should not be provided. Barriers that concern sport facilities should be provided. Based on the activity, barriers concerning required social context or required equipment should be suggested. Barriers concerning required expertise should not be suggested. Based on the profile, female-gender specific barriers should not be provided.

A total of 40 barriers meet these criteria and are provided as recommendations. Within this use case, someone cancelling is chosen as a barrier.

**Step 4: Coping Strategy Choice.** Based on the chosen barrier, coping strategies concerning motivation should not be suggested. Only coping strategy specific to the barrier “someone might cancel” should be suggested, as well as coping strategies that are always relevant.

Three coping strategies met these criteria and were provided as recommendations. Doing the activity without that person is chosen as a coping strategy.

## Case evaluation 5 Theo

Use case 5 is a 54 year-old male user called “Theo”. He is married, works part-time and has a high household income. He has no social preference and no preference for duration and has a beginner fitness level.

**Step 1: Activity Choice.** Based on the profile, this user should be suggested any activities that are not high intensity. No restrictions are given by the household income, social preference or duration preference.

38 activities meet these criteria and are provided as recommendations. Within this use case, “Swimming” is chosen as an activity.

**Step 2: Context Choice.** Based on the activity, a sports facility is recommended as the only possible location. The activity can be performed indoor and outdoor, either by oneself, with one additional person or in group. A minimum duration of 20 minutes is recommended.

Within this use case, an indoor Exercise Facility is chosen, the activity is planned alone and for 30 minutes total.

**Step 3: Barrier Choice.** Based on the context, barriers that are only relevant to outside activities should not be provided. Barriers that concern sport facilities should be provided. Based on the activity, barriers concerning required equipment should be suggested. Barriers concerning required social context or required expertise should not be provided. Barriers concerning the planned social context should not be provided. Based on the profile, female-gender specific barriers should not be provided.

A total of 38 barriers meet these criteria and are provided as recommendations. Within this use case, forgetting required equipment is chosen as a barrier.

**Step 4: Coping Strategy Choice.** Based on the chosen barrier, coping strategies concerning motivation should not be suggested. Only coping strategy specific to the barrier “forgetting material” should be suggested, as well as coping strategies that are always relevant.

Three coping strategies met these criteria and were provided as recommendations. Putting the material in sight as a visible reminder is chosen as a coping strategy.
